# Measurement of Cable Force through a Fiber Bragg Grating-Type Thin Rod Vibration Sensor and Its Application

**DOI:** 10.3390/s22208081

**Published:** 2022-10-21

**Authors:** Wanxu Zhu, Wei Teng, Fengrong Liu, Dongwen Wu, Yujiao Wu

**Affiliations:** 1 Guangxi Engineering Research Center of Intelligent Structural Material, Guilin University of Technology, Guilin 541004, China; 2 Guangxi Key Laboratory of Geotechnical Mechanics and Engineering, Guilin University of Technology, Guilin 541004, China; 3 Nanning Department of Civil and Surveying Engineering, The Airport Campus, Branch of Guilin University of Technology, Nanning 532100, China

**Keywords:** cable force monitoring, fiber grating, frequency method, cable-stayed bridge, resonance

## Abstract

The key to evaluating the health status of cable-stayed bridges lies in the accuracy of cable force measurement. When measuring the cable force using the conventional frequency method, the clearance between the bracing cable and the protective tube is typically disregarded. Moreover, due to their large size, existing vibration sensors are difficult to install into protective tubes for steel strand-type bracing cables to measure the cable force. To address the above difficulties, a type of thin rod vibration sensor only 5 mm in diameter was designed based on the high sensitivity of Fiber Bragg grating (FBG), and high-throughput data processing software for engineering calculation (EC) was self-developed. Then, the recognition principle of the thin rod vibration sensor was theoretically analyzed and a step-by-step tension test was carried out. The results demonstrated that the relative error of the cable force measured by the thin rod vibration sensor within 12.865 Hz was less than 5% and the sensitivity reached 28.7 pm/Hz, indicating its high measurement precision. Upon subsequent application of the thin rod vibration sensor to a monitoring test in the field, the relative error of the fundamental frequency between artificial and natural excitations was less than 4%. In addition, the error relative to both the theoretical frequency and the third-party sampling frequency was less than 5%, further verifying the accuracy and applicability for monitoring the cable force of bridges under natural excitation. Compared with the traditional cantilever FBG sensor, the improved sensor with supporting data processing software has the advantages of small cross-section, high reliability, and good sensitivity. The research results can provide a reference for the subsequent accurate measurement of cable force and the development of a supporting sensor data processing system.

## 1. Introduction

Structural health monitoring technology has become a current hot issue in the engineering field [1]. For cable-stayed bridges, cables are the main load-bearing components, and play the role of elastic support and force transmission [2]. Due to long-term vehicle load and wind and rain excitation, the stay cables are vulnerable to fatigue and corrosion [3], resulting in changes in cable force, which has a significant impact on the static characteristics and linearity of the bridge in the later period. Therefore, we evaluated the damage of a cable-stayed bridge by monitoring the change rule of cable force [4]. Recently, the cable force of stay cables has been re-measured by the oil pressure method, pressure sensor method, magnetic flux method, digital image method, and other measured frequency methods for the estimation of cable force by vibration both in China and overseas. While the oil pressure gauge method is simple and direct, the accuracy is low [5]. For the pressure sensor method [6], while the precision is high, the sensor has the disadvantage of large volume [7], even when advanced 3D printing technology is used to reduce the volume [8,9]. Moreover, the above two approaches are only applicable to during construction stage. Following a bridge’s abandonment, magnetic flux can be used to detect bridge cable defects [10] because of its good performance in detecting defects in steel wire ropes. In a study by Kim et al. [11,12,13], magnetic flux leakage (MFL) was used to diagnose fracture, corrosion, and compression damage to cables. In addition, a multi-stage pattern recognition method that can detect the internal and external damage of the main cable and combine artificial neural network (ANN) has been investigated. However, the magnetic flux method is susceptible to chemical corrosion leading to MFL [14], and the current size affects the electromagnetic force [15]. Du et al. [16] used two measurement methods based on digital image technology, namely, digital image processing (DIP) and digital image correlation (DIC). A camera captures single-point and multi-point images to identify the cable force, however, as shown by Jo et al. [17], it is vulnerable to fluctuations in external loads such as temperature and wind, and it is troublesome to measure the instantaneous tension of the cable. Wang et al. [18] noted that even if high-performance and expensive cameras are used, they may not provide sufficient resolution.

The vibration frequency method has been widely used in practical monitoring because of its high measurement accuracy, intuitive installation, and simple operation [19]. The equivalent effective length is used to expand the dual mode frequency method. For example, Fang et al. [20] used the lateral vibration equation of the cable and a practical formula for estimating the cable tension in a simple form using the bending stiffness. The equation of Ma et al. [21] has been revised to consider practical formulas for cable end anchorage devices and shock absorbers. Considering the influence of the bending stiffness, Fu et al. [22] proposed a cable force calculation method using the cable length and diameter with an error of less than 10%. Wang et al. [23] verified that the cable bridge deck interaction does not affect the low order frequency of the cable-stayed beam, and proposed its use to estimate the actual treatment method for cable forces.

Todd et al. [24] developed a cantilever beam-type FBG vibration sensor and theoretically analyzed its resonance frequency and sensitivity. Fuhr et al. [25] optimized a cantilever beam-type FBG vibration sensor and mounted it on a bridge to verify the reliability of the resonant frequency theory. Hu et al. [26] implemented the resonant frequency-to-cable force conversion according to the string vibration theory and employed this method to monitor the vibration characteristics of the bracing cables of Tongwamen Bridge. Furthermore, there have been numerous research studies on fiber grating vibration sensors. Zou et al. [27] proposed a cantilever vibration acceleration sensor with a maximum tilt angle fiber grating. Jia et al. [28,29,30] designed and introduced a fiber grating vibration sensor with a cantilever structure, and in order to improve the sensitivity of acceleration signal measurement, further developed an FBG acceleration sensor based on the combination of a cantilever and sliding bar. Li et al. [31] put forward a low-cost and high-sensitivity optical accelerometer assembled from a seismic mass, a leaf spring, and a sensing component based on a four-quadrant photodetector (QPD). Wang et al. [32] reported a MEMS acceleration sensor based on a dual-ended tuning fork resonator, which is suitable for low-frequency and low-g acceleration measurement. Zhao et al. [33] measured the string vibration frequency of suspension cables using the frequency method, and further developed a formula to indirectly quantify the cable force. Zhang et al. [34] constructed an FBG vibration sensor and a real-time supporting cable force monitoring system and applied them to the long-term remote real-time monitoring of the cable force of the Second Changjiang River Bridge in Wuhan, however, the data processing in this method is excessively tedious. Su et al. [35] designed an FBG vibration sensor based on the flexible hinge principle to extend the operating frequency range of their sensor and boost its sensitivity. Li et al. [36]. developed an acceleration sensor with a sensitivity of 2532.6 pm/g, placed a mass block in the middle, and directly connected it to the fibers at both ends, then converted the vibration at the mass block into the axial compression of fibers. However, this sensor possesses a large encapsulated cross-sectional area. Thus far, there has been an absence of efficient real-time processing software for the central wavelength data of fibers. When monitoring the cable force all FBG vibration sensors abide by the principle that a reciprocally vibrating mass block drives the cantilever beam of the embedded FBG to bend in order to capture the vibration frequency signal of the bracing cables. The cable force is then computed through the frequency method. The bracing cables of bridges are divided into two types, namely, steel strands and steel wires, of which steel strand-type bracing cables are normally assembled onsite and have clearance between their external protective tube and the steel strand. Large frequency measurement errors can be generated if vibration sensors are directly installed on the bracing cables. In addition, the mass block, which is of a large cross-sectional area, cannot be placed in such vibration sensors between the protective tube and the steel strand due to the tiny clearance spaces.

In this study, based on the engineering background of cable force monitoring of Xiangsizhou Bridge, we first developed a fiber grating-type thin rod vibration sensor with no mass block, and which as such can be placed inside the protective tube. Then, based on theoretical analysis of the structure, the frequency method was introduced to establish the relevant calculation formula of cable force and wavelength. Finally, the reliability and sensitivity of the sensor’s cable force monitoring were investigated through indoor repeated tensioning tests and field monitoring tests, and supporting engineering calculation (EC) software was developed for data processing. Compared with previous acceleration sensors, the new vibration sensor has a higher natural frequency. The sensor structure itself does not produce resonance or chirp, and has good sensitivity and stability. In addition, the research provides a reference for its future application in the fields of construction monitoring and post-operation health monitoring of cable-staying.

## 2. Design of FBG-Type Thin Rod Vibration Sensor

### 2.1. FBG Vibration Recognition Theory

**Figure 1:**
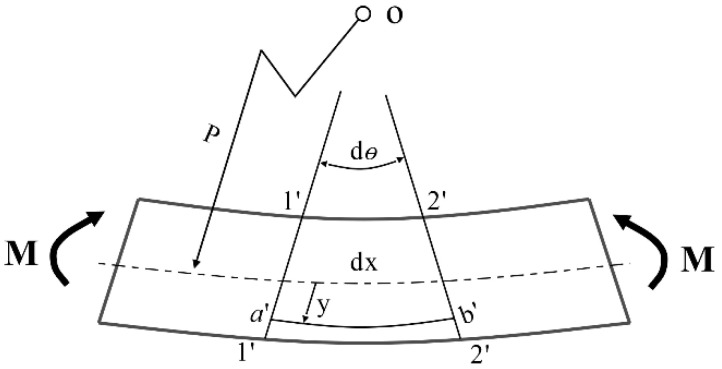
Schematic diagram of sensor geometric deformation.

When vibrating, the thin rod of this FBG-type thin rod vibration sensor is uniformly stressed as a homogeneous body (Figure 1). With a microelement randomly taken for analysis, the vibrating thin rod was subjected to axial flexural deformation, and two cross sections (1′-1′ and 2′-2′) with a distance of dx were rotated relative to the neutral axis. In addition, the cross sections 1′-1′ and 2′-2′ were extended and intersected at the center of curvature (point O) of the neutral layer. The neutral axis, the radius of curvature, and the included angle between the two cross sections were set as the X-axis, ρ, and dθ, respectively. The longitudinal positive strain at *y* is expressed as follows [37]:

(1) $$\Delta \varepsilon =\frac{\Delta L}{L}=\frac{\left(y+p\right)d\theta -\rho d\theta }{\rho d\theta }=\frac{y}{\rho }$$

In accordance with the elasto-optical effect of fibers, the strain directly results in a central wavelength shift of FBG [37]:

(2) $$\Delta \lambda =\left(1-\rho_{e}\right)\lambda \varepsilon_{x}$$

The following equation can then be obtained through Hook’s law and the axial balance of internal force:

(3) $$k\Delta x=E_{0}\varepsilon_{x}s$$

where *k*, Δx, $E_{0}$, $\varepsilon_{x}$, and *s* represent the material’s elastic coefficient, the expansion amount at any position on the X-axis, the elastic modulus, the strain along the X-axis, and the cross-sectional area, respectively.

**Figure 2:**
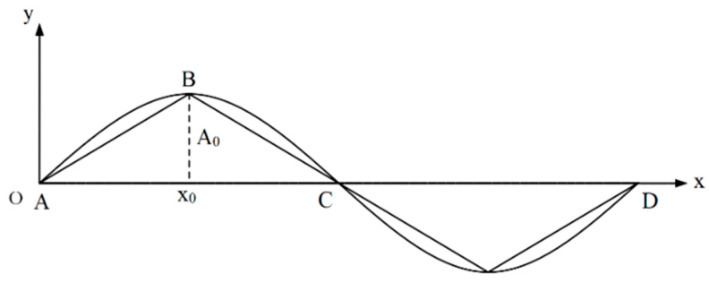
Schematic diagram of cable body vibration.

It can be observed from Figure 2 that when the cable body vibrates freely, the vibration signal is able to disperse into multiple sinusoidal signals with varying frequencies and amplitudes. The vibration signal may appear as a sinusoidal signal [29]:

(4) $$Y\left(t\right)=A_{0}\sin\left(\omega t+\varphi \right)$$

In this case, *Y*(*t*) represents the microelement shift along the Y-axis, *ω* denotes the circular frequency of microelement vibration, $A_{0}$ indicates amplitude, and *φ* indicates the initial phase of microelement vibration. Then,

(5) $${\left(x_{0}+\Delta x\right)}^{2}-x_{0}^{2}=Y^{2}\left(t\right)=A_{0}^{2}\sin^{2}\left(\omega t+\varphi \right)$$

By simultaneously solving Equations (3) and (5), the following equation can be acquired:

(6) $$\varepsilon_{x}=\frac{k\left(\sqrt{A_{0}^{2}\sin^{2}\left(\omega t+\varphi \right)+x_{0}^{2}}-x_{0}\right)}{E_{0}S}$$

Hence, the strain of the cable body is associated with the structural vibration frequency, with the latter being solved through the former, as demonstrated in Equation (7):

(7) $$\omega =\frac{\arcsin\sqrt{\frac{E_{0}^{2}S^{2}\varepsilon_{X}^{2}+2kx_{0}E_{0}S\varepsilon x}{A_{0}^{2}k^{2}}}-\varphi }{t}$$

According to the notion of sensitivity and Equation (7), the sensitivity *S* of this thin rod vibration sensor can be solved as follows [37]:

(8) $$S=\frac{\Delta \lambda }{\Delta f}=\frac{\left(\lambda_{i+1}-\lambda_{i}\right)t}{\left(2\pi \arcsin\sqrt{\frac{E_{0}^{2}S^{2}\varepsilon_{x}^{2}+2kx_{0}E_{0}S\varepsilon x_{x+1}}{A_{0}^{2}k^{2}}}-2\pi \arcsin\sqrt{\frac{E_{0}^{2}S^{2}\varepsilon_{x}^{2}+2kx_{0}E_{0}S\varepsilon x_{x}}{A_{0}^{2}k^{2}}}\right)}$$

where ω=2πf, in which f, Δλ, and Δf represent the frequency, the initial wavelength difference, and the fundamental frequency difference between two adjacent loading stages, respectively.

### 2.2. Fast Fourier Transform (FFT)

The FFT of two-dimensional (2D) continuous functions is introduced as follows [38]:

(9) $$F\left(\omega ,y\right)=\int_{-\infty }^{\infty }\int_{-\infty }^{\infty }f\left(\Delta \lambda ,t\right)e^{-j2\vartheta \left(w\Delta \lambda ,yt\right)}d\lambda dt$$

where f(Δλ,t) denotes the Fourier transform, *w* represents the frequency, and *y* indicates the amplitude. The continuous signal f(Δλ,t) is transformed into a 2D discrete signal f(k,l) according to the sampling frequency, where 0≪k≪M−1 and 0≪l≪N−1. Following this, the 2D inverse Fourier transform is expressed as follows:

(10) $$f\left(k,l\right)=\left(1/MN\right)\sum_{m=0}^{M-1}\sum_{n=0}^{N-1}F\left(f_{n},A\right)\exp\left[j2\vartheta \left(kf_{n}/M+lA/N\right)\right]$$

In other words, the discrete signal f(k,l) can be transformed into $F(f_{n},A)$, that is, FFT. Following the external excitation, the amplitude of the frequency $f_{n}$ identified by the sensor at the resonance point is significantly greater than that identified at a distance from the resonance point.

The cable force is converted by the vibration frequency method using the following formula [17,39]:

(11) $$T=\frac{4ml^{2}f_{n}^{2}}{n^{2}}$$

where *m*, *l*, and $f_{n}$ represent the linear density, the length of the bracing cable, and the n-order natural vibration frequency, respectively.

### 2.3. Structural Design of the Thin Rod Vibration Sensor

**Figure 3:**
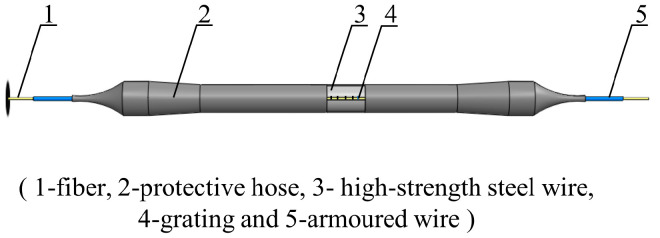
The schematic diagram of the fiber grating type thin rod vibration sensor.

**Figure 4:**
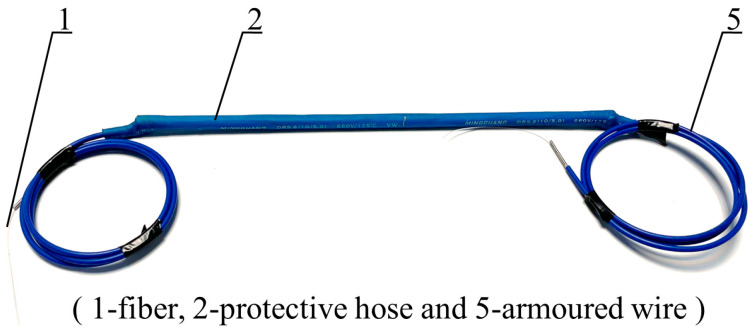
The real diagram of the fiber grating type thin rod vibration sensor.

The two most widely used specifications of steel strands in engineering are Φ 5 mm and Φ 7 mm. Through theoretical calculation according to Equation (8) and analysis of pre-experiment results, it was found that sensors with Φ 5 mm steel strands have better sensitivity. Based on the FBG vibration recognition theory and FFT, a 5 mm diameter FBG-type thin rod vibration sensor (Figure 3 and Figure 4) was developed. This thin rod vibration sensor is comprised of the following parts: No. 1 represents the optical fiber used for signal transmission; No. 2 refers to a protective hose used to prevent corrosion of the high-strength steel wire and damage to the fiber grating embedded in the slot; No. 3 represents the grating, which serves to identify the strain change in the vibration process; No. 4 represents high-strength steel wire, which is the base material of the sensor; and No. 5 represents the armored wire, which is used to protect the terminal lead wire from damage.

The fabrication process of the FBG thin rod vibration sensor was as follows: first, the high strength steel wire substrate with a length of 300 mm and a diameter of 5 mm was slotted along the axial direction. Second, the pre-tensioned optical fiber was placed in the slot of the substrate and the grating measuring point was positioned at the midpoint of the substrate. Third, the optical fiber was bonded to the high-strength steel wire substrate by adhesive and was let out at both ends. Fourth, substrate was sleeved with a protective hose slightly longer than itself, which was then heated to fit the high-strength steel wire substrate via heat shrinkage. Finally, the optical fiber was checked to ensure that the signal could be observed and met the requirements of no more than a slight change in wavelength when stationary and an obvious change in wavelength when vibrating.

The materials of the FBG-type thin rod vibration sensor are relatively common and inexpensive; the average production cost should be less than USD 50.

## 3. Frequency Response Test of FBG-Type Thin Rod Vibration Sensor

### 3.1. Test Design

**Figure 5:**
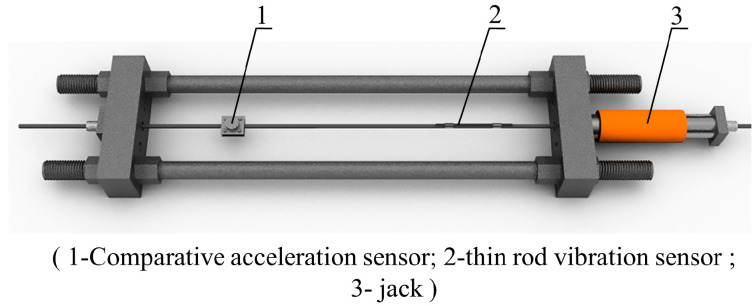
Tension test schematic.

**Figure 6:**
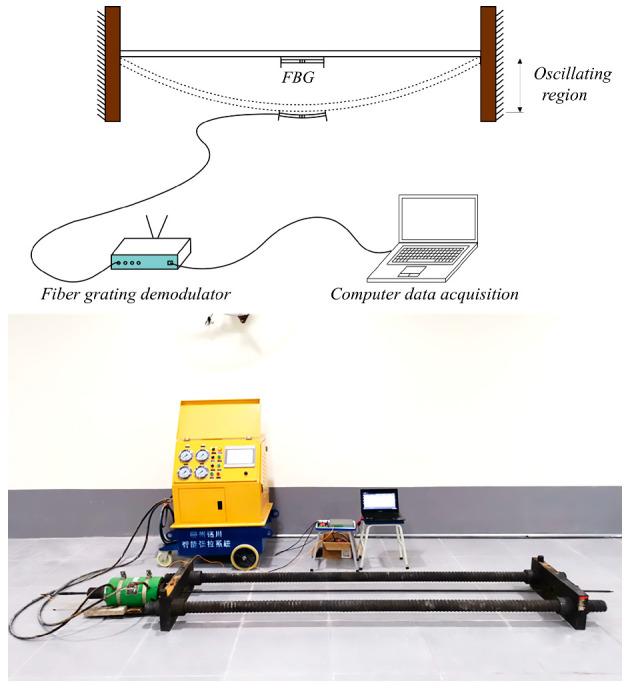
Vibration signal acquisition system schematic. and physical map.

**Figure 7:**
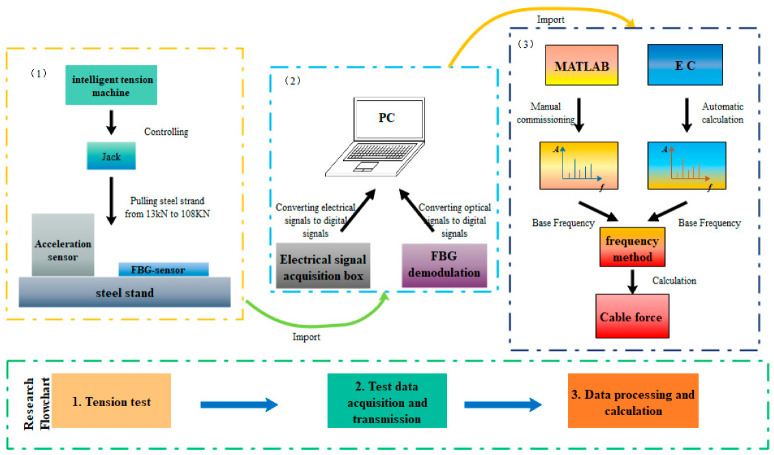
Schematic diagram.

The FBG-type thin rod vibration sensor, along with an acceleration sensor carrying a mass block for comparison, were mounted on the same pre-stressed steel strand, followed by the step-by-step tensioning of this steel strand (Figure 5) via an intelligent tension machine. The schematic diagram and an actual photo of the overall vibration signal acquisition system are displayed in Figure 6. Next, the grating measurement signal of the thin rod vibration sensor and the measurement signal of the acceleration sensor carrying a mass block were retrieved through a demodulator, converted into force values through FFT, and then compared to actual force values. The steel strand employed in the test was 12 m in length, 15.2 mm in diameter, 140 mm^2^ in cross-sectional area, and 260 kN in ultimate bearing capacity (P_n_). In addition, the 3 dB FBG sensor was 0.12 mm in bandwidth, 10 mm in optical grating length, and 99.64% in reflectivity. Moreover, the demodulator was a spectrometer (Agilent86142B) with a wavelength range of 1525–1565 nm, a sampling frequency of 100 Hz, a wavelength accuracy of 2.5 pm, and a resolution of 1 pm. Figure 7 shows the flow chart of the test method used during the whole article, including data collection, analysis, and theoretical calculation.

The frequency perception scheme implemented by the FBG-type thin rod vibration sensor was as follows. (1) The steel strand was pretensioned with a load of 0.2 P_n_ = 52 kN, which was sustained for 3 min, followed by unloading to 0.05 P_n_ = 13 kN. (2) Tensioning was performed incrementally from 0.05 P_n_ (13 kN) to 0.4 P_n_ (104 kN), with 13 kN of load applied at each step and a maximum tensioning rate of 100 MPa/min. (3) Data collection commenced after completing each step of tensioning and sustaining the load for 2 min. In each step, the reading of the demodulator and the force value displayed on the intelligent tension machine were collected three times, for a duration of 3 min each, followed by an interval of 2 min. (4) After 30 s of the data collection, a mallet was used to strike the center of the entire steel strand in a perpendicular direction. (5) Unloading was performed until 0.05 P_n_ after the third acquisition process at a step of 0.4 P_n_ was completed. (6) Steps 2–5 were repeated thrice, that is, tensioning and unloading were repeated three times.

### 3.2. Data Processing

**Figure 8:**
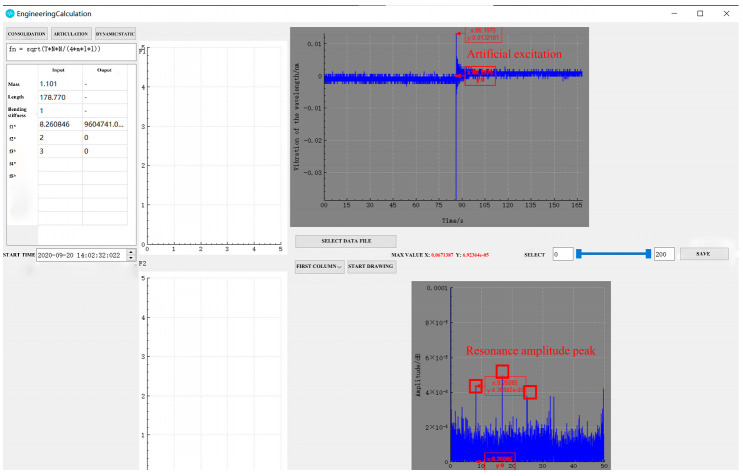
Data processing software interface.

The performance of a data processing module has a direct bearing on the measurement speed of the entire monitoring system. Based on the C++ programming language, an EC program (Figure 8) was developed that could directly convert the central wavelength reading continuously acquired for 3 min by the 100 Hz FBG demodulator into a time-domain diagram and a spectrum. This program primarily consisted of three modules: image drawing, function calculation, and image output and data storage. First, the data path saved by the demodulator was found, then a time-domain diagram with the central wavelength signal as the y-axis and time as the x-axis could be automatically drawn by clicking on the path. Meanwhile, FFT was automatically implemented to acquire the spectrum using Equations (9) and (10) as the function calculation basis. Then, the corresponding x-axis, namely, the n-order resonance frequency monitored by the sensor, could be acquired by clicking on the peak value. Moreover, the first-order resonance frequency of the bracing cable was determined through the peak value method and the frequency difference method, then automatically substituted into Equation (11) with parameters entered in advance to realize the rapid conversion of the cable force. Finally, the image was stored by clicking on it, and two images and the cable force were output together for later inquiry and deep analysis. It is noteworthy that during the operation process of the EC program any section of the sampling interval could be adjusted, with the spectrum redrawn correspondingly, allowing for greater potential in practical application.

### 3.3. Test Structure and Analysis

**Figure 9:**
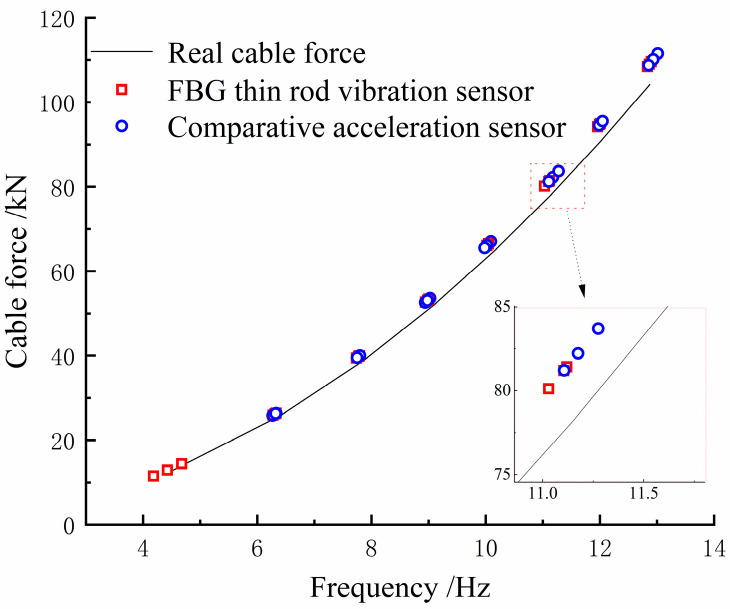
Comparison of cable force converted from fundamental frequency and actual value.

**Figure 10:**
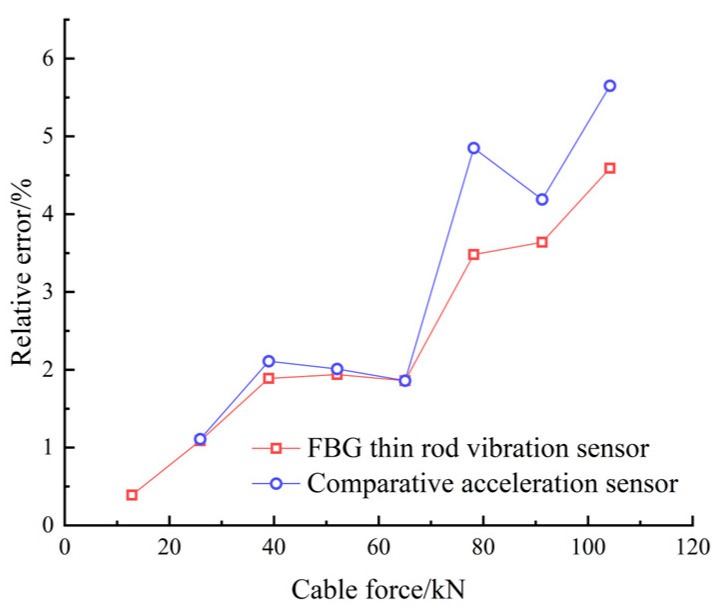
The error of the two sensors in identifying the actual cable force of the cable.

**Figure 11:**
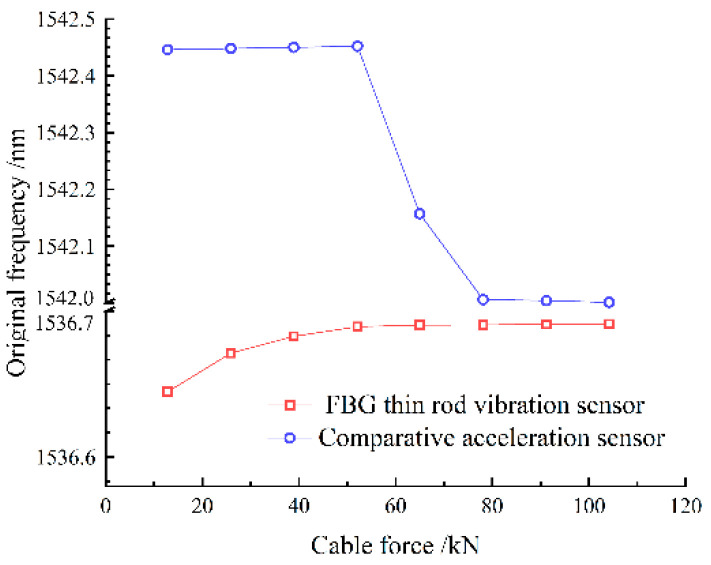
Slim rod induction meter graded loading initial wavelength of each stage.

**Figure 12:**
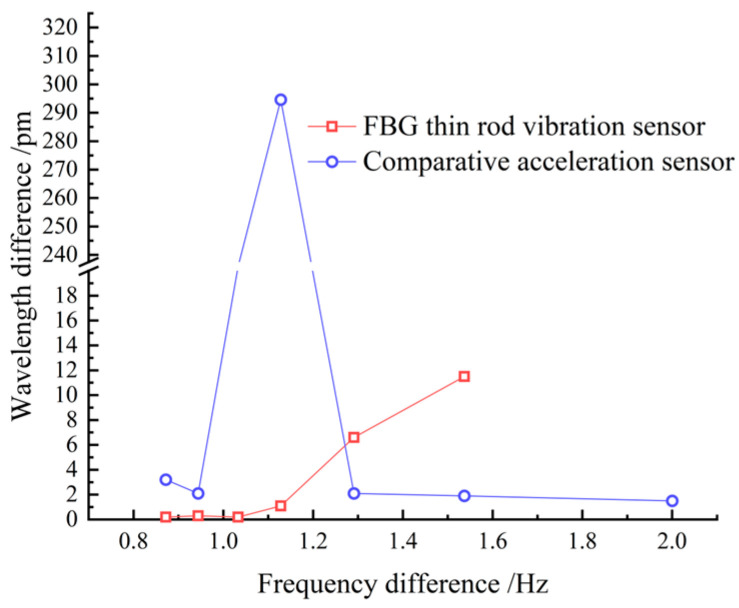
Sensitivity of the two sensors.

During the tensioning process, the cable vibrates, increasing the collection frequency noise. Therefore, data acquisition was performed only after each stage of tension, when the tension force and frequency are relatively stable. Combining Table 1 and Figure 9, it can be seen that both the sampling frequencies obtained by the thin rod vibration sensor and the control acceleration sensor, as well as the practical frequency, grew with the increase in the cable force, thus complying with Equation (11). However, the thin rod sensor was able to monitor the fundamental frequency (4.184 Hz), which could not be identified by the control acceleration sensor; hence, it had superior low-frequency recognition capabilities. The frequency corresponding to the actual force value can be back-calculated by the frequency method, meaning that the force and frequency of the cable are linear. The sensor collects when the cable force reaches the force value set for each stage and is stable and constant. As depicted in Figure 10, the relative errors of the cable forces measured by the two sensors to the actual cable force generally increased with the increase in the frequency, where the thin rod vibration sensor obtained a smaller relative error and met the practical engineering monitoring requirement specified in Mechanical Vibration and Shock—Experimental Determination of Mechanical Mobility (-ISO 7626-1:2011) [40], i.e., the error of sensors should be less than 5%. As shown in Figure 11 and Figure 12, when the tension force is 65 kN, the initial wavelength of the acceleration sensor decreases by 0.295 nm, indicating that the external excitation makes the sensor chirp during the monitoring process. In addition, for acceleration sensing near 10 Hz the sensitivity fluctuates significantly, with the maximum value being 294.6 pm/Hz, indicating that the fundamental frequency corresponding to the cable force is close to the inherent frequency of this acceleration sensor and the two resonate, which increases the error. Below 52.1 kN, the sensitivity of the thin rod vibration sensor was 28.7 pm/Hz. When the tension load exceeded 52.1 kN, the initial wavelength regained its position at 1536.69 nm and no longer increased. In this case, the sensor and strand may not have enough friction to prevent slippage. However, the error remains within 5%, and accuracy can be improved by changing the clamping mode. As a result, the thin rod sensor has a wider monitoring range and is less prone to resonance and chirp.

## 4. Engineering Application of FBG-Type Thin Rod Vibration Sensor

### 4.1. Project Profile

**Figure 13:**
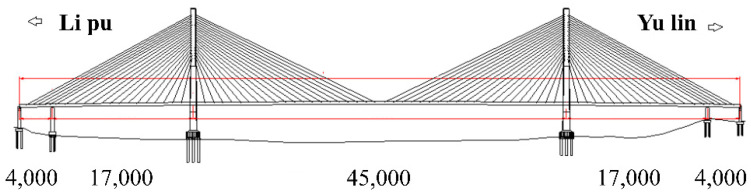
Schematic diagram of cable surface of Xiangsizhou Bridge.

As depicted in Figure 13, Xiangsizhou Bridge,1668 m in length, is a twin-tower double-cable-plane semi-floating cable-stayed bridge. The main tower is of a diamond-shaped structure. A reinforced concrete composite beam serves as the primary beam, and parallel steel strands are adopted as the bracing cables. In general, the natural frequency [41] of the bracing cable for a long-span bridge is approximately 0.5 Hz. To accurately measure the first-order natural frequency of the bracing cable, it is necessary to directly acquire the fundamental frequency signal of the steel strand-type bracing cable using a high-precision low-frequency vibration sensor, which can be placed in the small clearance between the protective tube and the steel strand with a small cross-sectional area. According to the indoor incremental tensioning test, the FBG-type thin rod vibration sensor demonstrated a smaller cross-sectional area and superior low-frequency recognition capability compared to the control acceleration sensor. Therefore, the FBG-type thin rod vibration sensor was utilized to acquire the fundamental frequency signal of the cable body in this project.

### 4.2. Field Monitoring

**Figure 14:**
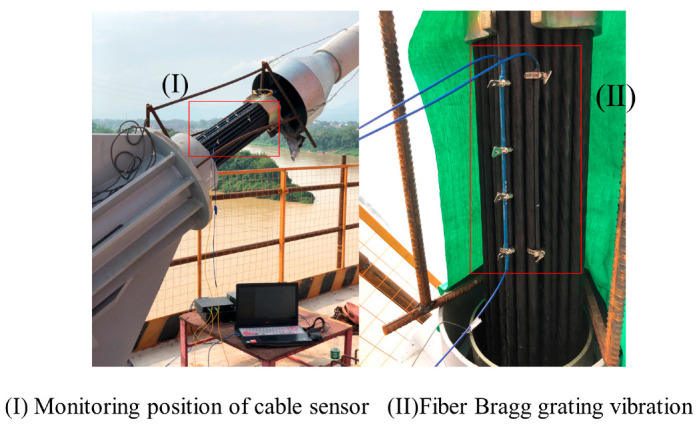
Position diagram of FBG thin rod vibration sensor.

With upstream midspan cables NB13 and NB14 on the north bank of Xiangsizhou Bridge serving as the monitoring objects, the thin rod vibration sensor was installed in the protective tube adjacent to the cable-beam anchor end, 1.5 m from the bridge deck (see (I) in Figure 14). As can be observed from (II) in Figure 14, the thin rod vibration sensor was directly coupled with the steel strand of the bracing cable by underlaying the hold hoop in two locations. Moreover, the vibration signals under natural and artificial excitations (knocking with a mallet) were acquired via spectrometer. The sampling lasted 300 s under natural excitation and 180 s under artificial excitation.

### 4.3. Monitoring Results and Analysis

The frequency signals acquired under the artificial excitation are exhibited in Table 2. The average frequency acquired simultaneously three times by sensors 1 and 2 on NB13 was 0.6226 Hz, while that acquired simultaneously by sensors 3 and 4 on NB14 was 0.6377 Hz. As per Equation (11), the average cable force of the former was calculated to be 47.34 kN, whereas that of the latter was calculated to be 53.35 kN. It can be found from Table 3 that for the frequency signals acquired under natural excitation, the average frequency acquired simultaneously by sensors 1 and 2 on NB13 for three repetitions was 0.6131 Hz, while that acquired simultaneously by sensors 3 and 4 on NB14 for three repetitions was 0.6189 Hz. According to Equation (11), the average cable force of the former was computed as 46.79 kN, whereas that of the latter was computed as 53.91 kN. Under both natural and artificial excitations the relative error of the cable forces measured by sensors 1 and 2 was 1.17%, while that of the cable forces measured by sensors 3 and 4 was 2.58%, indicating that the cable force monitoring results under two distinct excitation modes are quite approximate. Under natural excitation, therefore, the FBG-type thin rod vibration sensor was able to achieve the same monitoring effect as under the artificial excitation and accurately identify the cable force of the bracing cable. Table 4 presents the comparison results between the data acquired by the FBG-type thin rod vibration sensor under natural excitation and the monitoring data from the contractor (CCCC Second Harbor Engineering Co., Ltd., Shanghai, China). As seen in Table 4, the relative errors of the vibration frequencies on NB13 and NB14 to the theoretical frequency were 4.35% and 4.78%, respectively. In addition, the relative errors of the vibration frequencies on NB13 and NB14 to the third-party sampling frequency were 1.55% and 3.04%, respectively. Hence, the relative error of the frequency acquired by sensors when subjected to natural excitation compared to the theoretical frequency and to the sampling frequency were each less than 5%.

Overall, the small-volume FBG-type thin rod vibration sensor can be mounted within the protective tube of the bracing cable and directly coupled with the bracing cable. Moreover, this sensor can accurately monitor the low vibration frequency of the cable-stayed bridge, indicating its good low-frequency response performance. In addition, it is capable of accurately identifying the fundamental frequency of the bracing cable under natural excitation.

## 5. Conclusions

In this study, an FBG-type thin rod vibration sensor and a rapid data processing software EC were self-developed based on the FBG vibration recognition theory and the frequency method. According to this method, an indoor tensioning test and a field monitoring test on the frequency recognition of this sensor and the conversion of the cable force were carried out.

(1) The FBG-type thin rod vibration sensor with a 5 mm diameter overcomes the difficulty of conventional sensors, namely, that, they are difficult to install in the protective tube of the bracing cable. In addition, this sensor provides technical support for vibration sensors to be directly coupled with steel strand-type bracing cables.
(2) Within an indoor testing frequency range of 4–13 Hz, the relative error was always less than 2% under the fundamental frequency of 10 Hz, accompanied by a sensitivity of 28.7 pm/Hz. When the incremental tensioning cable force exceeded 52.1 kN, the measurement accuracy declined due to the relative slip between the steel strand and the sensor. However, the maximum error remained within 5%, and the relative error of the sampling frequency under the natural excitation was likewise less than 5%, which essentially matched the indoor test result. Hence, this sensor is of a relatively high accuracy.
(3) The EC data processing software, which is fast and inexpensive with a high sample throughput, substantially improved the efficiency of the cable force measurement system, thereby providing a reference for the development of real-time cable force monitoring sensors.

This paper studied the applicability of FBG thin rod vibration sensors in cable force monitoring through indoor and outdoor tests. To avoid the defects of the coupling method in indoor tests, we used an AQD19 Pneumatic Packaging Machine to strengthen the fastness of the coupling method. However, the outdoor tests do not account for the limitations of long-term data monitoring. Currently, the project is being monitored to provide further data support for the improvement and promotion of the application of this sensor in rope force monitoring technology.

## Figures and Tables

**Table 1:** Indoor cable tension test data.

Graded Loading Force (kN)	Theoretical Frequency (Hz)	Fiber Bragg Grating Thin Rod Vibration Sensor				Comparative Acceleration Sensor			
		1st Test Frequency (Hz)	2nd Test Frequency (Hz)	3rd Test Frequency (Hz)	Initial Wavelength (nm)	1st Test Frequency (Hz)	2nd Test Frequency (Hz)	3rd Test Frequency (Hz)	Initial Wavelength (nm)
12.8	4.533	4.676	4.427	4.492	1536.645	/	/	/	1542.446
25.9	6.449	6.331	6.311	6.713	1536.671	6.331	6.321	6.267	1542.448
38.9	7.923	7.775	7.737	8.333	1536.683	7.797	7.748	7.785	1542.450
52.1	9.146	8.946	8.983	9.629	1536.689	8.940	8.978	9.025	1542.452
65	10.200	10.020	10.020	10.747	1536.690	10.021	9.979	10.091	1542.157
78.1	11.205	11.030	11.120	11.870	1536.691	11.106	11.275	11.175	1542.006
91.2	12.101	11.960	11.993	12.830	1536.691	11.984	12.045	12.025	1542.004
104.2	12.934	12.900	12.832	13.757	1536.691	12.932	12.852	13.013	1542.001

**Table 2:** Frequency test results under artificial excitation.

Stay Cable Serial Number	Sensor Serial Number	1st Test Frequency (Hz)	The Second Test Frequency (Hz)	The Third Test Frequency (Hz)	Average Frequency (Hz)	Average Cable Force (kN)
NB13	1	0.6109	0.611	0.6114	0.6226	47.34
	2	0.6190	0.627	0.656		
NB14	3	0.6168	0.6159	0.6163	0.6377	55.35
	4	0.7735	0.5874	0.6168		

**Table 3:** Frequency test results under natural excitation.

Stay Cable Serial Number	Sensor Serial Number	1st Test Frequency (Hz)	The Second Test Frequency (Hz)	The Third Test Frequency (Hz)	Average Frequency (Hz)	Average Cable force (kN)
NB13	1	0.6146	0.6145	0.6103	0.6131	46.79
	2	0.6146	0.6145	0.6103		
NB14	3	0.6185	0.6205	0.6176	0.6189	53.91
	4	0.6185	0.6205	0.6176		

**Table 4:** Vibration frequency test results of stay cables.

Stay Cable Serial Number	Theoretical Frequency (Hz)	Monitoring Frequency (Hz)	Natural Excitation Frequency (Hz)	Relative Error of Natural Excitation and Theoretical Frequency (%)	Relative Error of Natural Excitation and Monitoring Frequency (%)
NB13	0.641	0.6226	0.6131	4.35%	1.55%
NB14	0.650	0.6377	0.6189	4.78%	3.04 %

## Data Availability

The dataset used in this study is included in the attachment.

## References

[1] Zheng R., Liu L., Zhao X., Chen Z.Q., Zhang C.Y., Hua X.G. (2018). Investigation of measurability and reliability of adhesive-bonded built-in fiber Bragg grating sensors on steel wire for bridge cable force monitoring. Measurement.

[2] Zhang L., Qiu G., Chen Z. (2021). Structural health monitoring methods of cables in cable-stayed bridge: A review. Measurement.

[3] Xu B., Dan D., Yu X. (2022). Real-time online intelligent perception of time-varying cable force based on vibration monitoring. Eng. Struct..

[4] Chen C.C., Wu W.H., Liu C.Y., Lai G.L. (2018). Diagnosis of instant and long-term damages in cable-stayed bridges based on the variation of cable forces. Struct. Infrastruct. Eng..

[5] Sun J.B., Zhao Z.Z., Zhao H.H. (2011). Measuring methods of cable tension in cable-stayed bridges. Adv. Mater. Res..

[6] Hao P., Yu C., Feng T., Zhang Z.H., Qin M.L., Zhao X., He H., Yao S. (2020). PM fiber based sensing tapes with automated 45° birefringence axis alignment for distributed force/pressure sensing. Opt. Express.

[7] Hong C.Y., Yuan Y., Yang Y.Y., Zhang Y.F., Zamir A.A. (2019). A simple FBG pressure sensor fabricated using fused deposition modelling process. Sens. Actuators A Phys..

[8] Ding Y.C., Xu T., Onyilagha O., Fong H., Zhu Z.T. (2019). Recent advances in flexible and wearable pressure sensors based on piezoresistive 3D monolithic conductive sponges. ACS Appl. Mater. Interfaces.

[9] Tang Z.H., Jia S.H., Zhou C.H., Li B. (2020). 3D printing of highly sensitive and large-measurement-range flexible pressure sensors with a positive piezoresistive effect. ACS Appl. Mater. Interfaces.

[10] Ni Y., Zhang Q., Xin R. (2021). Magnetic flux detection and identification of bridge cable metal area loss damage. Measurement.

[11] Kim J.W., Park S. (2018). Magnetic flux leakage sensing and artificial neural network pattern recognition-based automated damage detection and quantification for wire rope non-destructive evaluation. Sensors.

[12] Kim J.W., Kim J., Park S. (2019). Cross-sectional loss quantification for main cable NDE based on the BH loop measurement using a total flux sensor. J. Sens..

[13] Kim J.W., Tola K.D., Tran D.Q., Park S. (2019). MFL-based local damage diagnosis and SVM-based damage type classification for wire rope NDE. Materials.

[14] Qu Y., Zhang H., Zhao R.Q., Liao L., Zhou Y. (2019). Research on the method of predicting corrosion width of cables based on the spontaneous magnetic flux leakage. Materials.

[15] Jamali-Abnavi A., Hashemi-Dezaki H. (2021). Evaluating the electromagnetic forces on the electric arc furnaces’ power cables under various operation conditions. Electr. Power Syst. Res..

[16] Du W.K., Lei D., Bai P.X., Zhu F.P., Huang Z.T. (2020). Dynamic measurement of stay-cable force using digital image techniques. Measurement.

[17] Jo H.C., Kim S.H., Lee J., Lim Y.M. (2021). Sag-based cable tension force evaluation of cable-stayed bridges using multiple digital images. Measurement.

[18] Wang N.N., Ri K.W., Liu H., Zhao X.F. (2018). Structural displacement monitoring using smartphone camera and digital image correlation. IEEE Sens. J..

[19] Syamsi M.I., Wang C.Y., Nguyen V.S. (2022). Tension force identification for cable of various end-restraints using equivalent effective vibration lengths of mode pairs. Measurement.

[20] Fang Z., Wang J. (2012). Practical formula for cable tension estimation by vibration method. J. Bridge Eng..

[21] Ma T.Y. (2018). Research on Cable Force Measurement Based on Frequency Method for Cables in Complex Boundary Conditions.

[22] Fu Z.Q., Ji B.H., Wang Q.D., Wang Y.X. (2017). Cable force calculation using vibration frequency methods based on cable geometric parameters. J. Perform. Constr. Facil..

[23] Wang L.H., Zhang X.Y., Huang S., Li L.F. (2015). Measured frequency for the estimation of cable force by vibration method. J. Eng. Mech..

[24] Todd M.D., Johnson G.A., Althouse B.A., Vohra S.T. (1998). Flexural beam-based fiber Bragg grating accelerometers. IEEE Photonics Technol. Lett..

[25] Hu J. (2012). Research and Application of Jingyue Bridge Structural Health Monitoring System.

[26] Hu D.T., Guo Y.X., Chen X.F., Zhang C.R. (2017). Cable force health monitoring of Tongwamen bridge based on fiber Bragg grating. Appl. Sci..

[27] Zou X., Deng O., Luo B.B., Wu D.C., Zhao M.F., Zhong N.R., Song T., Jiang S.H., Xie L., Liu P. (2020). Research on the Performance of Cantilever Low Frequency Vibration Sensor Based on Thin-diameter Excessively Tilted Fiber Grating. Acta Photonica Sin..

[28] Jia Z.A., Zhao X.F., Yang K.Q., Liu Y.G., Fan W. (2020). Fiber Bragg grating vibration sensor based on ‘E’ Beam Structure. Optoelectron. Laser.

[29] Jia Z.A., Zhang X., Li K., Fan Q.G. (2019). Research on a Cantilever Fiber Bragg Grating Vibration Sensor. Piezoelectric Acousto-Opt..

[30] Jia Z.A., Zhang X., Li K., Fan Q.G. (2018). Research on a new type of sliding-rod fiber grating vibration sensor. Optoelectron. Laser.

[31] Li R.J., Lei Y.J., Chang Z.X., Zhang L.S., Fan K.C. (2018). Development of a high-sensitivity optical accelerometer for low-frequency vibration measurement. Sensors.

[32] Wang S.D., Wei X.Y., Zhao Y.L., Jiang Z.D., Shen Y.J. (2018). A MEMS resonant accelerometer for low-frequency vibration detection. Sens. Actuators A Phys..

[33] Zhao X.F., Han R.C., Ding Y.B., Yu Y., Guan Q.H., Hu W.T., Li M.C., Ou J.P. (2015). Portable and convenient cable force measurement using smartphone. J. Civ. Struct. Health Monit..

[34] Zhang D., Li W., Guo D., Hu J., Luo P., Jiang D.S. (2007). Real-time monitor system of bridge-cable force based on fbg vibration sensor and its application. Chin. J. Sens. Actuators.

[35] Su L., Zhang X.T., Lv P.T., Sun M.Y., Wang C.H., Chen C., Zhang T.Y. (2020). Research on a fiber grating low frequency vibration sensor with large range and high sensitivity. Infrared Laser Eng..

[36] Li T.L., Tan Y.G., Han X., Zheng K., Zhou Z. (2017). Diaphragm based fiber Bragg grating acceleration sensor with temperature compensation. Sensors.

[37] Liu H.L., Zhu Z.W., Zheng Y., Liu B., Xiao F. (2018). Experimental study on an FBG strain sensor. Opt. Fiber Technol..

[38] Jang M.J., Chen C.L., Liu Y.C. (2001). Two-dimensional differential transform for partial differential equations. Appl. Math. Comput..

[39] Li T.L., Tan Y.G., Zhou Z. (2016). A fiber Bragg grating sensing-based micro-vibration sensor and its application. Sensors.

[40] (2011). Mechanical Vibration and Shock–Experimental Determination of Mechanical Mobility–Part 1: Basic Terms and Definitions, and Transducer Specifications.

[41] Tang G.M. (2006). Analysis of Mounted Resonant Frequency of Piezo-Electric Acceleration Sensor and Its Application.

